# Surgical treatment of lone atrial fibrillation

**DOI:** 10.1590/S1516-31802010000600010

**Published:** 2010-12-02

**Authors:** Shi-Min Yuan, Hua Jing, Leonid Sternik

**Affiliations:** I MD, PhD. Postdoctoral Researcher, Department of Cardiothoracic Surgery, Jinling Hospital, School of Clinical Medicine, Nanjing University, Nanjing 210002, Jiangsu Province, People's Republic of China.; II MD. Professor and Head, Department of Cardiothoracic Surgery, Jinling Hospital, School of Clinical Medicine, Nanjing University, Nanjing 210002, Jiangsu Province, People's Republic of China.; III MD. Consultant Surgeon, Department of Cardiac and Thoracic Surgery, The Chaim Sheba Medical Center, Tel Hashomer 52621, Israel.

**Keywords:** Arrhythmias, cardiac, Atrial fibrillation, Cardiac surgical procedures, Cardiopulmonary bypass, Minimally invasive surgical procedures, Arritmias cardíacas, Fibrilação atrial, Procedimentos cirúrgicos cardíacos, Ponte cardiopulmonar, Procedimentos cirúrgicos minimamente invasivos

## Abstract

Currently, off-pump video-assisted thoracoscopic epicardial pulmonary vein isolation offers an attractive alternative to on-pump Maze procedures for surgical treatment of lone atrial fibrillation. Nevertheless, on-pump Maze procedures through a mid-sternotomy approach still play an important role in patients with lone atrial fibrillation on many occasions, especially in patients with failed percutaneous pulmonary vein alone. The aim of this article was to give a brief review of the surgical strategies for treating lone atrial fibrillation, and present the possible indications for on-pump Maze procedures through a mid-sternotomy approach.

## RESUMO

Atualmente, isolamento epicárdico da veia pulmonar com cirurgia torácica vídeo-assistida sem circulação extracorpórea oferece uma alternativa atraente para procedimento de Maze com circulação extracorpórea no tratamento cirúrgico da fibrilação atrial solitária. No entanto, procedimento de Maze com circulação extracorpórea através de uma meio-esternotomia ainda desempenha papel importante em pacientes com fibrilação atrial isolada em várias ocasiões, especialmente em pacientes com falha da veia pulmonar percutânea isoladamente. O objetivo do artigo é fazer uma breve revisão de estratégias para o tratamento cirúrgico fibrilação atrial isolado e apresentar as possíveis indicações de procedimento de Maze com extracorpórea, através de uma abordagem meio-esternotomia.

## INTRODUCTION

Lone atrial fibrillation (LAF) was firstly termed before the modern era of echocardiography, based on a cohort of young patients with atrial fibrillation in the absence of clinical evidence of cardiovascular disorders.^1^ The patients’ ages and associated comorbidities, and the echocardiographic evidence of mild abnormalities, with regard to the concept of LAF, remain subjects to be resolved.^2^ LAF patients, and especially those with chronic LAF, are at increased risk of embolic complications and higher mortality rates.^3^ Aspirin rather than warfarin has been recommended for patients with LAF because of their relatively low risk of thromboembolism and stroke.^2^ Left atrial catheter ablation has emerged as a successful method for eliminating atrial fibrillation, but it may cause esophageal perforation at the level of the left atrium, with mediastinal soiling, which requires endoscopic placement of a removable esophageal stent in order to seal off the esophagomediastinal fistula.^4^ Percutaneous ablation has given rise to some major complications, including pulmonary vein stenosis, thromboembolism and atrioesophageal fistula.^5^ For LAF patients who are resistant to medical therapies, off-pump video-assisted thoracoscopic epicardial pulmonary vein isolation (PVI) offers an attractive alternative to on-pump Maze procedures for surgical treatment of LAF. However, on-pump Maze procedures through a mid-sternotomy approach still play an important role in patients with LAF on many occasions, especially in patients with failed percutaneous pulmonary vein alone.

## METHODS

A literature search was conducted in the Lilacs, PubMed, Embase and Cochrane Library databases using the search terms “lone atrial fibrillation” and “surgery”.

A manual search of abstracts of articles was made to identify those relating to this topic.

## RESULTS

The results are presented in Table 1.

**Table 1.** Results from literature search in the major medical databases

Database	Search strategies	Results		
Cochrane	“lone atrial fibrillation” AND “surgery”	3 found	3 related	3 randomized controlled trials
Embase (Excerpta Medica databases) (1983-2010)		89 found	83 related	6 case reports 2 case-control studies 2 prospective cohort studies 23 retrospective cohort studies 2 retrospective comparative studies 10 randomized controlled trials 9 basic research studies 17 reviews 12 others
Lilacs (Literatura Latino-Americana e do Caribe em Ciências da Saúde)		0 found		
PubMed		126 found	116 related	2 case series 7 case reports 24 prospective cohort studies 4 prospective comparative studies 18 basic research studies 23 retrospective cohort studies 4 retrospective comparative studies 22 reviews 6 randomized controlled trials 6 others

## DISCUSSION

### Concept of LAF

LAF was defined as atrial fibrillation in patients younger than 70 years old with no clinical or echocardiographic evidence of primary cardiac disorders, hypertension or occult thyrotoxicosis.^3,5,6^ LAF was categorized into three types: chronic paroxysmal, persistent or permanent.^7^ A study based on a large patient population showed that the incidence of LAF was 2.13% (76/3623), and the mean age of the patients with LAF was 44.2 ± 11.7 years at first diagnosis.^7^ The duration of LAF may vary from a few hours to several days, and may even last for over a week in occasional cases. The frequency of intermittent LAF may be one to three times a year.^8^ LAF patients with mitral regurgitation have been found to present greater mitral valve annular area and left atrial area than do those without mitral regurgitation, thus indicating the potential correlation between mitral regurgitation and left atrial dilation and the corresponding mitral annular dilation.^9^ Spontaneous action potential depolarization and a lower threshold for the action potential associated with genetic mutations of the cardiac sodium channel (SCN5A), specific only to the phenotype of atrial fibrillation, were identified recently.^10^

### Surgical indications

Atrial fibrillation now remains the most frequent cause of embolic events, stroke and death.^11^ Catheter ablation may carry a risk of stroke associated with the thrombus produced in relation to an increase in tissue impedance and an elevation in local temperature. Left atrial appendage exclusion has been found to be impossible with catheter-based ablation. It has often been associated with postoperative atrial tachyarrhythmia that might be caused by incomplete and non-transmural ablation lines.^12^ The classic indications for surgical treatment are intolerance to prolonged anti-arrhythmic or anticoagulant medication, medically refractory arrhythmia^5^ and failure of catheter-based ablation on one or more occasions; or situations in which the patients are not candidates for catheter-based ablation.^13,14^ Moreover, LAF patients with comorbidities such as transient ischemic attack and cerebrovascular accident are also surgical candidates.^15^ Nowadays, LAF patients who are refractory to medical treatments and for whom surgical treatment is preferable, either at their own request or their physicians’ instigation, are candidates for novel ablation procedures.^14^

### Classifications of the Maze procedures

Most of the operations for LAF have been performed along with other cardiac surgical procedures and mainly through a median sternotomy approach. In contrast to the standard procedures, closed-chest thoracoscopic PVI has been developed as a solution for the technical complexity and surgical invasiveness.^16^ Based on the maneuver techniques, Maze procedures have been classified into two types: surgical procedures (Cox-maze/cut-and-sew, and mini-partial maze) and energy-based procedures using radiofrequencies, microwaves, cryotherapy, ultrasound or laser, etc.^17^

There are two types of ablation: endocardial and epicardial. Endocardial ablation, which is done during cardioplegic arrest, may easily result in transmural lesions by using different energy sources and technical devices. Epicardial techniques have made it possible to perform surgical maneuvers on a beating heart, thus simplifying the procedure through having a full set of devices available. However, epicardial fat and the heat sink effect of the flowing endocardial blood might be obstacles to effective ablation for transmural effects.^18,19^

### On-pump mid-sternotomy Maze procedure

There is some debate on the mechanisms of atrial fibrillation with regard to whether macro-reentry circuits in the atria or the drivers of atrial fibrillation within the pulmonary veins produce the effect.^17^ The Maze I procedure was started primarily for LAF patients. Since 1995, the Maze III procedure has been performed, usually in associated with a mitral valve procedure.^20^ Cox-Maze III surgery was developed based on the concept of macro-reentry circuits in the atria.^17^ It has been recognized as the gold standard for surgical treatment of LAF cases with limited stroke complications. However, its complexity and technical difficulty have hampered the spread of this procedure.^21^ The Cox-Maze IV procedure was devised to isolate the right and left pulmonary veins as two islands, but preserve the right atrial appendage. This procedure is performed with the aid of cardiopulmonary bypass via median sternotomy or right thoracotomy,^22^ and it may be carried out on a beating heart.^21^ Pulmonary vein antrum isolation may be helpful in increasing the success rate and reducing the risk of complications, based on the hypothesis that an atrial fibrillation driver predominates within the pulmonary veins.^23^ The conversion rate can reach 98% in patients receiving pulmonary vein antrum isolation.^23^ However, this approach is technically complex and invasive.^24^ Moreover, the postoperative pacemaker implantation rate and postoperative atrial fibrillation rate have been found to be high.^25^

Energy source approaches including radiofrequencies, microwaves and cryoablation were developed as alternatives to Cox-Maze III surgery. Continuous linear transmural atrial lesions made by these energy sources may produce heart block.^25^ With a unipolar probe, energy disperses in multiple directions, and thus may damage the adjacent structures, such as the esophagus and coronary arteries.^17^ With a bipolar probe, the energy produces a transmural lesion, with both epicardial and endocardial features, within a shorter ablation time.^17^

On-pump Cox Maze III surgery has resulted in a sinus rhythm rate of 79-95%, but this procedure may occasionally be associated with major complications of sinus node dysfunction after a mean follow-up of 4.8 years.^6,26^ Nevertheless, the Maze procedure using a mid-sternotomy approach still plays an important role among patients with LAF on several occasions, especially among those with failed percutaneous PVI. The on-pump Maze procedure may achieve a sinus rhythm in 80-90% of the patients with paroxysmal atrial fibrillation, and in over 30% of those with persistent atrial fibrillation.

### Off-pump Maze procedures

Most of the surgical procedures have been carried out with the aid of cardiopulmonary bypass, and therefore they were performed endocardially.^27^ The minimally invasive procedure using bipolar radiofrequency devices was developed to achieve bilateral PVI and excision of the left atrial appendage through bilateral minithoracotomy and thoracoscopy, namely, an epicardial approach.^24,27^ This could avoid sternotomy or rib-spreading thoracotomy, along with cardiopulmonary bypass,^12^ and it has minimized the morbidity caused by surgical treatment of atrial fibrillation.^24^ PVI alone might be effective for most patients with paroxysmal atrial fibrillation and some of the patients with persistent atrial fibrillation.^24^ However, the right-sided approach has the drawback of not dealing with the left atrial appendage, and the uncertainty of the transmural nature of the lesions caused by microwave energy on the beating heart.^5^

Alternative energy resources have made it possible to perform PVI ablation off-pump and epicardially via thoracoscopic or robotic-assisted routes. The Flex 10^TM^ microwave ablation device is a popular energy source available for PVI. Complete ablation by microwaves has been obtained in animal models, without major complications.^28^ In clinical patients, a sinus rhythm has been seen in up to 79.5% of the patients, with a low mortality rate of 2-3%.^29-32^ Video-assisted beating-heart bilateral PVI or Marshall dissection on veins, with left atrial appendage closure, has proven to be successful by the use of radiofrequencies, such that 80-100% of the patients recover a normal sinus rhythm.^5,25,33^ There was no mortality in these cases, but some patients presented complications: pacemaker requirement in 5%, phrenic nerve palsy in 3%, hemothorax in 3%, transient ischemic attack in 1% and pulmonary embolism in 1%.^33^ In a recent report, UltraCinch^TM^ with high-intensity focused ultrasound (HIFU) was used for 15 LAF patients, among whom 11 were paroxysmal and four were persistent. This technique achieved a sinus rhythm in 40% of the patients. Endocardial radiofrequency re-ablation was required in 40% of the patients, and two major complications occurred, including one case of late tamponade and one case of bleeding during surgery.^34^ In addition, laser can be an effective tool for endocardial ablation, in terms of effectiveness of isolation and transmural effects, as shown in a sheep model.^35^

Bisleri et al.^16^ did not observe any complications relating to bipolar radiofrequency use in six patients with off-pump video-assisted PVI, and all six patients achieved a sinus rhythm within the six-month observation period. Video-assisted bilateral PVI with endoscopic stapling of the left atrial appendage has achieved an atrial fibrillation-free rate of 91.3% after three months of follow-up.^12^

## CONCLUSIONS

Currently, the Maze procedure for LAF is very suitable for patients with previous cardiac surgery and previous catheter ablation.^12^ It does not lose its function as an alternative to anti-arrhythmic medical treatment, long-term anticoagulation, electrical cardioversion or catheter-based ablation (evidence level = 2A; grade of recommendation = B).^12,16^ In the near future, off-pump PVI with additional interatrial lesions and left atrial appendage exclusion, or hybrid approaches incorporating both surgical and percutaneous techniques, will be favored for surgical treatment of LAF (evidence level = 3A; grade of recommendation = A).^17^ Nonetheless, the importance of open-chest Maze cannot be overlooked (evidence level = 5; grade of recommendation = D).

## References

[1] Evans W, Swann P (1954). Lone auricular fibrillation. Br Heart.

[2] Gollob M, Manning WJ, Hart RG Lone atrial fibrillation. UpToDate.

[3] Scardi S, Mazzone C, Pandullo C (1999). Lone atrial fibrillation: prognostic differences between paroxysmal and chronic forms after 10 years of follow-up. Am Heart J.

[4] Bunch TJ, Nelson J, Foley T (2006). Temporary esophageal stenting allows healing of esophageal perforations following atrial fibrillation ablation procedures. J Cardiovasc Electrophysiol.

[5] Sagbas E, Akpinar B, Sanisoglu I (2007). Video-assisted bilateral epicardial pulmonary vein isolation for the treatment of lone atrial fibrillation. Ann Thorac Surg.

[6] Jessurun ER, van Hemel NM, Defauw JA (2000). Results of maze surgery for lone paroxysmal atrial fibrillation. Circulation.

[7] Jahangir A, Lee V, Friedman PA (2007). Long-term progression and outcomes with aging in patients with lone atrial fibrillation: a 30-year follow-up study. Circulation.

[8] Larsen HR Lone atrial fibrillation. The Afib Report.

[9] Kihara T, Gillinov AM, Takasaki K (2009). Mitral regurgitation associated with mitral annular dilation in patients with lone atrial fibrillation: an echocardiographic study. Echocardiography.

[10] Li Q, Huang H, Liu G (2009). Gain-of-function mutation of Nav1.5 in atrial fibrillation enhances cellular excitability and lowers the threshold for action potential firing. Biochem Biophys Res Commun.

[11] Grandmougin D, Tiffet O (2007). Video-assisted thoracoscopic epicardial ablation of left pulmonary veins for lone permanent atrial fibrillation. Interact Cardiovasc Thorac Surg.

[12] Pappone C, Radinovic A, Manguso F (2008). Atrial fibrillation progression and management: a 5-year prospective follow-up study. Heart Rhythm.

[13] Vicol C, Eifert S, Kur F, Reichart B (2005). Minimally invasive off-pump pulmonary vein isolation to treat paroxysmal atrial fibrillation. Thorac Cardiovasc Surg.

[14] Damiano RJ, Shoenberg JM (2009). Surgical treatment of lone atrial fibrillation 2009. 25th Annual Cardiology Conference at Lake Louise.

[15] Melby SJ, Zierer A, Lubahn JG (2008). Normal quality of life after the cox maze procedure for atrial fibrillation. Innovations Phila Pa.

[16] Bisleri G, Manzato A, Argenziano M, Vigilance DW, Muneretto C (2005). Thoracoscopic epicardial pulmonary vein ablation for lone paroxysmal atrial fibrillation. Europace.

[17] Jahangiri M, Weir G, Mandal K, Savelieva I, Camm J (2006). Current strategies in the management of atrial fibrillation. Ann Thorac Surg.

[18] Follis F, Wernly J (1994). The endocardial versus the epicardial approach: still a controversy? J Thorac Cardiovasc Surg.

[19] Hemmer W, Bohm JO (2007). Zukunftsvisionen und neue Technologien in der chirurgischen Behandlung des Vorhofflimmerns [New developments for surgical ablation of atrial fibrillation]. Herzschrittmacherther Elektrophysiol.

[20] McCarthy PM, Gillinov AM, Castle L, Chung M, Cosgrove D (2000). The Cox-Maze procedure: the Cleveland Clinic experience. Semin Thorac Cardiovasc Surg.

[21] Shen J, Bailey M, Damiano RJ (2009). Surgery for Lone Atrial Fibrillation: Present State-of-the-Art. Innovations Phila Pa.

[22] Lall SC, Melby SJ, Voeller RK (2007). The effect of ablation technology on surgical outcomes after the Cox-maze procedure: a propensity analysis. J Thorac Cardiovasc Surg.

[23] Khaykin Y, Marrouche NF, Saliba W (2004). Pulmonary vein antrum isolation for treatment of atrial fibrillation in patients with valvular heart disease or prior open heart surgery. Heart Rhythm.

[24] Khargi K, Keyhan-Falsafi A, Hutten BA (2007). Surgical treatment of atrial fibrillation: a systematic review. Herzschrittmacherther Elektrophysiol.

[25] Suwalski P, Suwalski G, Wilimski R (2007). Minimally invasive off-pump video-assisted endoscopic surgical pulmonary vein isolation using bipolar radiofrequency ablation - preliminary report. Kardiol Pol.

[26] Hemels ME, Gu YL, Tuinenburg AE (2006). Favorable long-term outcome of Maze surgery in patients with lone atrial fibrillation. Ann Thorac Surg.

[27] Puskas J, Lin E, Bailey D, Guyton R (2007). Thoracoscopic radiofrequency pulmonary vein isolation and atrial appendage occlusion. Ann Thorac Surg.

[28] Addis A, Vanosi G, Manasse E (2007). An experimental sheep model used to develop an ablation procedure for chronic atrial fibrillation. Surg Endosc.

[29] Pruitt JC, Lazzara RR, Ebra G (2007). Minimally invasive surgical ablation of atrial fibrillation: the thoracoscopic box lesion approach. J Interv Card Electrophysiol.

[30] Pruitt JC, Lazzara RR, Dworkin GH (2006). Totally endoscopic ablation of lone atrial fibrillation: initial clinical experience. Ann Thorac Surg.

[31] La Meir M, De Roy L, Blommaert D, Buche M (2007). Treatment of lone atrial fibrillation with a right thoracoscopic approach. Ann Thorac Surg.

[32] Koistinen J, Valtonen M, Savola J, Airaksinen J (2007). Thoracoscopic microwave ablation of atrial fibrillation. Interact Cardiovasc Thorac Surg.

[33] Beyer E, Lee R, Lam BK (2009). Point: Minimally invasive bipolar radiofrequency ablation of lone atrial fibrillation: early multicenter results. J Thorac Cardiovasc Surg.

[34] Klinkenberg TJ, Ahmed S, Ten Hagen A (2009). Feasibility and outcome of epicardial pulmonary vein isolation for lone atrial fibrillation using minimal invasive surgery and high intensity focused ultrasound. Europace.

[35] Doll N, Suwalski P, Aupperle H (2008). Endocardial laser ablation for the treatment of atrial fibrillation in an acute sheep model. J Card Surg.

